# Potential of animal models for advancing the understanding and treatment of pain in Parkinson’s disease

**DOI:** 10.1038/s41531-019-0104-6

**Published:** 2020-01-06

**Authors:** Yazead Buhidma, Katarina Rukavina, Kallol Ray Chaudhuri, Susan Duty

**Affiliations:** 1 King’s College London, Institute of Psychiatry, Psychology & Neuroscience, Wolfson Centre for Age-Related Diseases, Guy’s Campus, London, SE1 1UL UK; 2 Institute of Psychiatry, Psychology & Neuroscience at King’s College and King’s College Hospital NHS Foundation Trust, London, UK; 3 Parkinson’s Foundation Centre of Excellence, King’s College Hospital, London, UK

**Keywords:** Parkinson's disease, Animal disease models

## Abstract

Pain is a commonly occurring non-motor symptom of Parkinson’s disease (PD). Treatment of pain in PD remains less than optimal and a better understanding of the underlying mechanisms would facilitate discovery of improved analgesics. Animal models of PD have already proven helpful for furthering the understanding and treatment of motor symptoms of PD, but could these models offer insight into pain in PD? This review addresses the current position regarding pain in preclinical models of PD, covering the face and predictive validity of existing models and their use so far in advancing understanding of the mechanisms contributing to pain in PD. While pain itself is not usually measured in animals, nociception in the form of thermal, mechanical or chemical nociceptive thresholds offers a useful readout, given reduced nociceptive thresholds are commonly seen in PD patients. Animal models of PD including the reserpine-treated rat and neurodegenerative models such as the MPTP-treated mouse and 6-hydroxydopamine (6-OHDA)-treated rat each exhibit reduced nociceptive thresholds, supporting face validity of these models. Furthermore, some interventions known clinically to relieve pain in PD, such as dopaminergic therapies and deep brain stimulation of the subthalamic nucleus, restore nociceptive thresholds in one or more models, supporting their predictive validity. Mechanistic insight gained already includes involvement of central and spinal dopamine and opioid systems. Moving forward, these preclinical models should advance understanding of the cellular and molecular mechanisms underlying pain in PD and provide test beds for examining the efficacy of novel analgesics to better treat this debilitating non-motor symptom.

## Clinical features of pain in Parkinson’s disease

Pain is a debilitating non-motor symptom (NMS) occurring from the prodromal to palliative stages of Parkinson’s disease (PD).^1,2^ The prevalence of PD pain reported in epidemiological studies varies between 68% and 85%; nevertheless, it remains under-reported and is often not considered in clinical consultations.^3,4^

PD patients may experience various pain syndromes. Using the King’s Parkinson’s Disease Pain Scale (KPPS), the first validated PD-specific pain scale, which addresses localisation, intensity and frequency of pain, together with its association to motor fluctuations or musculoskeletal pain, the syndrome of spontaneous pain related to PD (SPPD) can be subdivided.^5^ The subdivided patterns include musculoskeletal pain, chronic pain (central or visceral), fluctuation-related pain, nocturnal pain, orofacial pain, discoloration/oedema/swelling and radicular pain. Recently, the King’s PD Pain Questionnaire (KPPQ) has been derived from the KPPS and validated. This screening tool allows for the direct declaration of the pain each patient experiences.^6^ Alternatively, quantitative sensory testing (QST) has also been extensively used in PD patients.^7–9^

The pathogenesis of PD pain is complex; specific neurodegenerative changes of both dopaminergic and nondopaminergic pathways may cause alterations in different dimensions of the experience of pain in PD patients.^10^ Although not consistent in all cases, numerous clinical studies reported reduced thermal, electrical, cold or mechanical pain thresholds in PD patients, reflective of hypersensitivity (Table 1). Interestingly, this seems to be independent of the presence of a clinical pain syndrome^11–14^ potentially indicating sub-clinical alterations to pain-related pathways in some patients.

**Table 1:** Changes in nociceptive thresholds in Parkinson’s patients.

Patient groups		Nociceptive	Thresholds		Ref
	Electrical	Thermal	Cold	Mechanical	
SPPD patients vs. control	n/a	n/a	↓	n/a	^85^
	↓	↓	n/a	n/a	^13^
	↓	n/a	n/a	↑	^11^
	n/a	↓	n/a	NS	^9^
	n/a	↓	n/a	n/a	^21^
No SPPD patients vs. control	n/a	↓	n/a	↓	^86^
	↓	n/a	n/a	n/a	^15,33^
	n/a	↓	n/a	n/a	^8,22,87–89^
	n/a	↓	n/a	↓	^7^
	↑	↑	n/a	n/a	^12^
	↓	↓	n/a	n/a	^13^
	↓	n/a	n/a	↑	^11^
	n/a	↓	n/a	NS	^9^
No SPPD patients vs. SPPD patients	n/a	↓	n/a	n/a	^24^
	NS	n/a	n/a	NS	^11^
	n/a	n/a	NS	n/a	^14^
	n/a	↓	n/a	NS	^9^
	n/a	↓	n/a	n/a	^21^
	NS	NS	n/a	n/a	^13^

Table 1 shows comparative changes in nociceptive thresholds in PD patients with and without SPPD (Spontaneous Pain in Parkinson’s Disease). ↓ = a reduction in thresholds indicating hypersensitivity, ↑ **=** an increase in thresholds indicating hyposensitivity, *n/a* test not performed, *NS* no significant difference

Neurodegenerative changes in both nigral dopaminergic and extra-nigral non-dopaminergic pathways (cholinergic, noradrenergic, and serotonergic) have been implicated in PD related changes in nociceptive processing.^10,15,16^ These changes are thought to be accompanied by changes in pain processing at the level of the spinal cord as well as alterations in peripheral transmission and sensory-discriminative processing, perception and interpretation of pain.^10,16^

The occurrence of pain in PD may be further impacted by genetic associations. For example, one study found an increased reporting of unexplained pain in patients that carried glucocerebrosidase (GBA) mutations (58%) compared to non-carriers (10%),^17^ while another found pain more likely to be a presenting symptom in GBA mutation carriers (10.3%) versus non GBA carriers (3.0%).^18^ However, others found no difference in either the levels of pain reporting or the likelihood of pain being listed as the presenting symptom between carriers and non-carriers.^19^ More clarification is therefore awaited on the potential link between GBA mutations and pain. Abnormalities of nociceptive processing reportedly also occur in PINK1 (gene encoding a mitochondrial serine/threonine- protein kinase) mutation carriers, although in this instance the mutation appears to lead to hypoalgesia when compared to non-PD controls,^20^ rather than the hyperalgesia noted in sporadic PD cases. Future investigation into the reason behind this switch in phenotype may be informative in relation to the pathophysiology of pain in PD.

With regards to current treatment, some reports indicate that SPPD is often neglected and insufficiently dealt with. Importantly, pain in PD patients requires distinction between pain directly related to the pathogenic process of PD and pain that arises secondary to comorbidity of PD.^5^

The management of SPPD is often through maintenance of stable bioavailability of dopaminergic drugs. For example, L-DOPA alone has been shown to alleviate SPPD and the hypersensitivity in PD patients^14,21,22^ while rotigotine transdermal patch, safinamide (a monoamine oxidase type B inhibitor given as an add-on therapy to L-DOPA) and intrajejunal L-DOPA infusion therapy also provide beneficial effects on pain sensations in PD patients.^10^ However, other studies report limited to no benefit of L-DOPA treatment against pain thresholds^9,15^ and no qualitative improvement in neuropathic pain.^23^ Furthermore, the dopamine agonist apomorphine fails to alter either pain thresholds or pain-induced cerebral processing in PD patients,^24^ supporting the involvement of additional, non-dopaminergic pathways in SPPD as noted above.^10,15,16^ Consistent with this, there are a number of efficacious non-dopaminergic and non-pharmacological interventions for alleviating pain in PD: duloxetine (a selective serotonin and noradrenaline reuptake inhibitor); botulinum toxin (for dystonic pain); oxycodone/naloxone for use in severe PD related pain; deep brain stimulation (DBS) of the subthalamic nucleus (STN) to regulate firing in this pathologically overactive nucleus.^10,25^ Conversely, the use of other analgesics such as tricyclics and atypical antipsychotics has been met with limited success.^26,27^ Indeed, pain is not adequately addressed or treated in 30.2% of an unselected clinic population of PD patients across several centres in Europe.^28^

From the above, it is apparent that treatment of pain in PD remains less than optimum and that our understanding of the origin and cause of some pain-related symptoms in PD is far from complete. One way in which advances in both the understanding and treatment of pain in PD may be made is through extensive pre-clinical investigations. However, to enable this, reliable animal models are required. To date, there has been no systematic review of animal models of pain in PD from which to gain confidence that such studies are a realistic prospect in this area.

## Preclinical models of pain in Parkinson’s disease

From the outset, it is important to note that we are not considering here animal models of pain *per se*. The existence of good models in the pain field is itself subject to ongoing debate and outside of the scope of this review.^29,30^ Here, we are specifically interested in whether, and how, animals can be used to model pain in PD. As such, our attention is focused solely on existing animal models of PD and how effectively they recapitulate what is seen with respect to pain in the clinical setting.

Animal models of PD provide a controlled means to assess behavioural changes, cellular dysfunction, neurochemical alterations and other neural mechanisms that may contribute to disease pathogenesis and pathophysiology in humans. There are many different models available, spanning from invertebrates to non-human primates and from reversible, transient pharmacological models to stable transgenic models. As comprehensively reviewed elsewhere, each model has its own strengths and weaknesses in terms of replicating the motor deficits and other key hallmarks of PD such as dopaminergic neuron degeneration.^31^ Some of the neurotoxic models in particular have stood the test of time and have helped progress our understanding of the pathophysiology and treatment of the motor aspects of the disease. However, the utility of animal models for exploring non-motor symptoms (NMS) of PD is less well explored. As succinctly reviewed, existing models do express some of the NMS,^32^ but, while cognitive and neuropsychiatric signs were discussed in some detail in this review, there was only passing mention of pain-related signs. Thus, it is timely to review the currently available options in terms of animal models for pain in PD.

Any favourable animal model should display the following features: construct validity, whereby similar pathogenesis to the disease is replicated as evidenced, for example, by oxidative stress, inflammation and complex I inhibition; face validity, whereby relevant symptoms, biochemistry and pathology to the human condition are exhibited; predictive validity whereby the model discriminates between clinically effective and ineffective therapeutic strategies. How much we can rely on these features to strengthen support for animal models of pain in PD needs careful consideration. On the one hand, it is almost impossible to achieve good construct validity given that, as discussed above, the underlying basis for pain in PD is not yet well established. Nevertheless, the models should display good construct validity for PD itself. As this is already established for all models discussed below,^31^ construct validity will not be considered further here. On the other hand, face validity, reflecting a pain-like state in animals, should be achievable and is of paramount importance. Finally, predictive validity should be determinable to some extent, considering there exist clinical examples of treatments effective against some forms of pain in PD, such as DBS of the STN or treatment with either L-DOPA or duloxetine.^33–38^

Studies investigating pain in animal models of PD have so far almost all been conducted in rodents: no data are available regarding pain phenotypes in non-human primate models of PD or those constructed in multicellular model organisms like zebrafish, *C. elegans* or drosophila. In the remainder of this review, we discuss the various rodent models of pain in PD, considering how well each exhibit face and predictive validity and how they have helped progress the understanding of pain in PD. Ultimately, it is hoped that one or more of these models might provide an accepted testbed for use in the search for novel analgesics to better treat pain in PD.

## Assessing nociception in rodents

While pain cannot be directly measured in rodents, as this requires a subjective component, sensory-discriminative aspects of pain (nociception) can be quantified and is the readout taken in animal studies of pain in PD. Nociception in rodents can be measured in several ways to delineate the different modalities of sensation that may be affected. Studies usually involve testing thermal (hot and cold), mechanical and chemical thresholds to assess if either allodynia (reduced nociceptive thresholds to non-noxious stimuli) or hyperalgesia (reduced nociceptive thresholds to noxious stimuli) are present. Heat thresholds can be measured with the hotplate, tail flick, Hargreaves or hot water bath test, as detailed in recent reviews,^39,40^ whereby latency to withdrawal of the paw/tail from the heat source is measured. Mechanical thresholds are measured via manual or electronic von Frey filaments and the Randall–Selitto test where the force required to cause withdrawal of the paw after application is measured.^41,42^ Cold response is normally measured with the acetone or cold plate test either by counting the number of paw flicks/licks after the acetone is applied to the paw or withdrawal latency of the paw from the cold plate, respectively.^43^ Finally, chemical nociceptive responses are assessed using capsaicin, with measurement taken of the time spent grooming/scratching the injected site.^44^ Although it is not possible to measure SPPD with these paradigms, the analysis of nociceptive thresholds provides good face validity, as PD patients experience reduced thresholds in numerous modalities, regardless of whether they report SPPD (Table 1). While it is possible to measure ongoing pain of a potentially spontaneous nature in rodents, using tests such as conditioned place preference, to demonstrate preference for an analgesic versus saline treatment,^45^ such approaches have not yet been applied to animal models of PD.

## Acute pharmacological models

The earliest in vivo models used for PD research were generated using pharmacological agents, reserpine and haloperidol.

Reserpine works by blocking the vesicular monoamine transporter and causing a subsequent transient depletion of central and peripheral monoamines including dopamine, 5-hydroxytryptamine (5-HT; serotonin) and noradrenaline, without concurrent neurodegeneration. In this respect it is considered a crude model of PD but nevertheless one that was instrumental in the discovery of L-DOPA.^46,47^ Reserpine treatment causes reduced mechanical and thermal nociceptive thresholds in rodents,^48,49^ indicative of good face validity for pain in PD. The antinociceptive effects of duloxetine in this model,^50^ further support an involvement of reduced serotonergic and noradrenergic signalling in the mechanical and thermal hypersensitivity. However, these pharmacological effects are not totally in line with clinical outcomes, casting some doubt on the predictive validity of this model. Thus, while duloxetine treatment led to a reduction in the number of SPPD being reported in patients, there were no changes in thermal thresholds, measured using QST, before and after treatment.^35^ It will be interesting to determine whether duloxetine reverses the reduced mechanical threshold noted in PD patients as this would then strengthen the predictive validity of the reserpine model. Overall, the reserpine model supports the importance of monoaminergic signalling for normal pain processing but does not help tease out the specific pathways involved in PD related pain. Furthermore, since reserpine treatment in rats (1 mg/kg s.c., once daily for three consecutive days) is also used to model fibromyalgia, a condition associated with widespread chronic pain,^51^ reserpine treatment cannot be considered a faithful model for pain in PD.

Haloperidol is another agent used to model PD in rodents. Haloperidol acts by reversibly blocking dopamine D_2_ receptors and the striatal blockade results in catalepsy that is considered a crude model of PD.^31^ Regardless, since haloperidol is widely shown to have analgesic properties,^52^ it can be completely ruled out as a potential model to study pain in PD.

## Neurodegenerative models

A key weakness in the above pharmacological models is their lack of PD-related pathology. However, there are many other chemical or toxin-induced models of PD that do exhibit degeneration of dopaminergic neurons in the substantia nigra pars compacta (SNc) and subsequent reduced striatal dopamine content. Described here under the collective heading of neurodegenerative models, it is clear some of these present promising models for exploring pain in PD.

## MPTP

MPTP (1-methyl-4-phenyl-1, 2, 3, 6-tetrahydropyridine) has long been used to generate animal models of PD that have proved instrumental in advancing both our knowledge and therapeutic approach to the treatment of PD.^53,54^ Following systemic dosing, MPTP crosses the blood-brain-barrier and, after conversion into 1-methyl-4-phenylpyridinium (MPP+) by astrocyte-derived monoamine oxidase, is taken up via dopamine transporters (DAT) into dopaminergic neurons. MPP + binds to complex I in the mitochondrial matrix disrupting oxidative phosphorylation and leading to downstream apoptosis and bilateral neurodegeneration.^55,56^ Use of this toxin is restricted to mice and non-human primates, since rats are resistant to systemic MPTP administration.^57^

To date, only two studies in mice (none in primates) have investigated nociceptive changes in the MPTP model. MPTP-treated mice appear to exhibit decreased heat, chemical and mechanical induced nociceptive thresholds^58,59^ which supports good face validity. This hypersensitivity is evident at day 7 post MPTP injection but has resolved by day 14, hindering this model’s utility for chronic pain investigations.^58^ In terms of treatment, these early threshold reductions were partially reversible with L-DOPA administration,^58,59^ supporting potential for good predictive validity, given the aforementioned efficacy of dopaminergic treatment against some clinical pain symptoms.^33^ Some mechanistic insight has already been drawn from the MPTP model. When hypersensitivity was evident, 7-days post MPTP injection, Rosland et al.^58^ found reduced dopamine levels in the spinal cord, but no alterations in 5-HT or noradrenaline. By day 14 post-MPTP, when the hypersensitivity had spontaneously resolved, 5-HT levels were now increased. These findings suggest that central dopaminergic changes have a knock-on effect at the level of the spinal cord, resulting in hypersensitivity which can be subsequently reversed perhaps by a compensatory increase of activity from the intact serotonergic systems in the CNS.^58,59^

## Rotenone and lipopolysaccharide

Other agents that have been used to induce PD-related neurodegeneration in rodents include rotenone and lipopolysaccharide.

Rotenone is a pesticide which, like MPTP, readily crosses the blood-brain barrier following systemic injection and inhibits complex I of the mitochondrial respiratory chain, inducing oxidative stress^60,61^ and subsequent pathology that is not confined to dopaminergic neurons.^62^ However, in the only nociception study in this model, rotenone did not alter mechanical thresholds,^63^ thus potentially ruling it out as a model to study pain in PD.

Lipopolysaccharide (LPS) is a bacterial endotoxin that activates glial cells to induce an inflammatory response in the host. Given the intimate link between microgliosis and dopaminergic cell loss in PD,^64^ LPS has been used to model the inflammatory aspects of PD. Following stereotaxic injection into the SNc, LPS triggers increased cytokine release and oxidative stress, with accompanying nigrostriatal tract degeneration.^65–67^ This model therefore has the potential to provide insight into the contribution of nigral neuroinflammation to pain in PD. However, nociceptive tests have thus far not been conducted in this model.

## 6-OHDA

One of the most widely used rodent models of PD is generated by stereotaxic administration of 6-hydroxydopamine (6-OHDA) into the medial forebrain bundle, the striatum or the SNc. 6-OHDA is taken up by DAT and the noradrenaline reuptake transporter, though selectivity for DAT can be achieved by coadministration of desipramine. Inside cells, 6-OHDA inhibits complexes I and IV of the mitochondrial respiratory chain and causes oxidative stress and neuroinflammation leading ultimately to neurodegeneration and motor impairment in the form of akinesia.^31^ Most commonly, 6-OHDA is administered unilaterally to develop a hemi-parkinsonian model that can bear full or partial nigrostriatal tract lesions depending on combinations of the dose and site of administration. This not only prevents the high mortality rate observed in bilateral full lesion models, but also means that the lesioned (ipsilateral) and intact (contralateral) hemispheres of the brain can be compared as well as side-specific behaviours. Notably, nigrostriatal lesioning causes motor impairment in the limbs on the side contralateral to the brain lesion. This is important to note because nociceptive tests require mobility in the test paw which must be accounted for when gauging the effectiveness of 6-OHDA-treated rodents for modelling pain.

The 6-OHDA model has been the most extensively explored in relation to modelling pain in PD. A range of studies, primarily conducted in rats which due to their size are easier to observe, have reported hypersensitivity to heat, mechanical, chemical or cold stimuli, as summarised in Table 2. These observations support good face validity of the model.

**Table 2:** Changes in nociceptive thresholds in 6-OHDA lesioned rats.

Rat Breed	Lesion location	Lesion type	Time when tests performed (weeks post-lesion)		Nociceptive	tests		Hind paw displaying change	Ref
				Heat	Mechanical	Chemical	Cold		
Wistar	Left MFB	Unilateral	1, 4 & 12	n/a	↓	n/a	n/a	Both	^90^
	Left MFB	Unilateral	3	n/a	n/a	↓	n/a	Ipsilateral	^91^
	Right MFB	Unilateral	2 & 4	↓	n/a	n/a	n/a	n/d tail immersion	^92^
	left SNc	Unilateral	2	↓	↓	↓	↓	Both	^75^
	Right STR	Unilateral	4	↓	↓	↓	n/a	Contralateral	^93^
	Left STR	Unilateral	1, 2 & 3	↓	↓	n/a	n/a	Both	^73^
Sprague-Dawley	Right MFB	Unilateral	2	↓	↓	n/a	n/a	Both	^73^
	Right MFB	Unilateral	3	↓	↓	n/a	n/a	Contralateral	^76^
	MFB	Bilateral	2	n/a	↓	↓	n/a	Both	^74^
	MFB	Bilateral	2	n/a	↓	n/a	n/a	Both	^94^
	SNc	Bilateral	1, 2, 3 & 4	↓	↓	n/a	n/a	Both	^77^
	Right STR	Unilateral	5 & 6	↑	n/a	↓	n/a	Contralateral	^95^
	Left STR	Unilateral	1, 2 & 3	↓	↓	↓	n/a	Both	^96^

Table 2 summarises the outcome of all nociceptive studies performed in 6-OHDA lesioned rats. ↓ = a reduction in thresholds, ↑ = an increase in thresholds, n/a = test not performed, n/d = readout not determinable. *MFB* medial forebrain bundle, *SNc* Substantia nigra pars compacta, *STR* striatum

These nociceptive changes have been observed as early as 1-week post lesion and persist for at least 12 weeks suggesting stable alterations, or persistent plasticity, underpin the changes. In most cases, the threshold changes are seen bilaterally, in both the ‘PD-affected’ (contralateral) and unaffected (ipsilateral) paws. This tells us firstly that the decreased latency to withdraw the paw from the stimulus is not simply reflecting motor impairment, since the ipsilateral paw has no impairment whatsoever. Secondly, this supports bilateral supraspinal involvement at the level of the brainstem that is controlled by upstream dopaminergic systems.

There is already strong evidence supporting a good level of predictive validity of the 6-OHDA rat for modelling pain in PD. For example, the reduced mechanical thresholds have been shown to return to baseline upon administration of L-DOPA,^68^ consistent with the fluctuations in SPPD and reversal of pain in patients upon L-DOPA administration.^14,21,22,33^ Furthermore, non-pharmacological interventions that are efficacious in the clinic such as the afore-mentioned DBS of the STN^69^ are also back translated into this model. Thus, DBS in the STN restores both mechanical and heat nociceptive thresholds of 6-OHDA lesioned rats back to control levels.^70,71^ This outcome may reflect reversal of electrophysiological changes that have been recorded in the STN of 6-OHDA lesioned rats in response to peripheral stimulation.^72^ Interestingly, the antinociceptive effect of DBS is amplified by co-administration of duloxetine^73^ yet treatment with duloxetine alone fails to normalise mechanical and heat thresholds in this model, contrary to what is seen in patients.^35^ Moreover, apomorphine, which was ineffective in patients,^24^ has been shown to reverse hypersensitivity in this model.^68^ It is clear therefore that predictive validity is strong, but that extra caution should be applied to avoid identifying false positives.

Given its reasonable face and predictive validity, the 6-OHDA rat model is likely to prove useful for investigations aimed at gaining mechanistic insight into the hypersensitivity seen. There is certainly plentiful evidence favouring involvement of the dopaminergic systems, considering the above noted restoration of nociceptive thresholds following L-DOPA treatment. Furthermore, the analgesic efficacy of L-DOPA may stem from activation of dopamine D_2_ receptors (DRD2) since bromocriptine, a selective DRD2 agonist, also restores mechanical thresholds back to control level 15 min after intraperitoneal injection in the 6-OHDA rat model.^74^ Additionally, replenishment of dopamine bioavailability through grafting of chromaffin progenitor cells into the striatum, restores normal nociceptive thresholds to thermal, mechanical and chemical stimuli, in a DRD2 dependent manner.^75^ However, it is unlikely that the nociceptive changes are solely controlled by dopaminergic systems since the beneficial effects of chromaffin progenitor cell grafts could be blocked and even reversed by naltrexone, an opioid antagonist,^75^ supporting a role for enhanced opioid transmission in this analgesic action post dopamine supplementation. This is consistent with the analgesic benefits provided by oxycodone/naloxone in PD patients with severe pain^25,27^ and is further supported by the findings of bilateral reductions in enkephalins and μ-opioid receptors and increased excitability of lamina V wide dynamic range neurons in the spinal cord of unilaterally 6-OHDA lesioned rats which expressed mechanical and heat hypersensitivity.^68,76^

Serotonergic involvement in the nociceptive hypersensitivity is also implicated. For example, 6-OHDA lesioned rats with nociceptive hypersensitivity show reduced numbers of 5-HT neurons in the rostral ventromedial medulla (RVM) and in the dorsal horn of the spinal cord,^77^ suggestive of reduced 5-HT transmission in these pain related regions. The reduced mechanical and thermal thresholds were acutely attenuated by intra-RVM administration of citalopram to boost 5-HT levels, further implicating serotonergic system dysfunction in these sensory changes. However, conversely, lesioning of serotonergic neurons in the RVM of 6-OHDA rats caused a partial reversal of nociceptive hypersensitivity,^77^ implying elevated, rather than reduced, 5-HT transmission in the RVM was behind the sensory changes. This latter finding marries closely with the notion of 5-HT signalling from the RVM facilitating pain transmission in the descending pathways during the development of persistent pain^78^ and highlights the need for further investigation into the role of 5-HT in nociceptive hypersensitivity.

Taken together, these results suggest that the mechanisms modulating nociceptive thresholds in the 6-OHDA lesion rat model of PD involve dopaminergic, serotonergic and opioid pathways, as reflected clinically.^14,35,79^ Given this close correlation between the 6-OHDA model and known clinical picture, further exploration in this model may help advance our understanding of the pathophysiology of pain symptoms and provide an ideal platform to test potential therapeutics.

## Genetic models

With the identification of more than 13 loci in 9 different genes associated with familial forms of PD,^80^ it is not surprising that numerous transgenic models of PD with abnormal production of PD-related proteins such as α-synuclein, parkin, PINK1, DJ-1, LRRK2 or UCHL1 have emerged into the research arena.^31^ However, very few of these models have yet been studied in relation to nociceptive changes and those that have display little in the way of changes. For example, overexpression of human SNCA gene in mice does not alter thermal thresholds,^81^ while mice transgenic for leucine-rich repeat kinase 2 (LRRK2;^R1441G^ PARK8 mutants) display no reduction in mechanical or chemical thresholds.^82,83^ These findings argue against any face validity of these particular transgenic models and suggest that neither of these gene abnormalities is linked to development of pain in PD. Whether other genetic mutations contribute to the pain phenotype in animals remains to be seen, although on the basis of clinical findings, PINK1 transgenic animals would be expected to exhibit nociceptive hyposensitivity, rather than hypersensitivity.^20^

Aside from familial PD-related genes, the GBA gene (which encodes glucocerebrosidase: a lysosomal enzyme involved in glucosylceramide to ceramide conversion) has raised interest.^84^ As noted earlier, PD patients heterozygous for GBA report an increase in SPPD.^18^ No preclinical investigations have yet been published looking at the specific mutations seen in PD, though our unpublished data fail to demonstrate any reductions in mechanical and heat thresholds in the GBA (D409V/WT) mouse at either six or twelve months of age. Future studies in animals bearing different GBA mutations might reveal different behaviours and shed further light on the potential usefulness of these models.

## Conclusion

We are clearly in possession of rodent models of PD which display a good level of both face and predictive validity for pain in PD. The reserpine-treated rat model is an acute, reversible model that will not lend itself to longitudinal studies and, without any pathology, the specific pathways responsible for the heightened sensory response cannot be teased out. Nevertheless, this model could provide a rapid testbed for the screening of novel analgesics with a monoaminergic component. In contrast, the MPTP-treated mouse and 6-OHDA-lesioned rat models look more promising and both show modest to good face and predictive validity. While the MPTP-treated mouse model offers a limited time window for potential analgesic testing given that the hypersensitivity resolves, the 6-OHDA-lesioned rat provides a stable model with persistent hypersensitivity against which to test novel analgesics over a longer-term. Both models should facilitate investigations into the mechanisms behind the hypersensitivity and in this context the resolving nature in MPTP-treated mice could be very informative if paralleled by resolving of changes in the brain or spinal cord of these animals. Without doubt these models are only that – models of pain in PD. As far as is currently known, they only replicate one aspect of pain in PD – the reduced sensory thresholds. However, as new ways to measure holistic pain in animals become established,^30^ there is scope for further enhancing the face validity and hence utility of these models. With analgesic provision for tackling pain in PD being itself modest, there is much hope pinned on establishing good animal models of pain in PD. It is anticipated these models will not only help to inform us of the potential cellular and molecular mechanisms underlying pain in PD but that they might also provide test beds for examining the efficacy of novel analgesics to better treat this NMS and improve the quality of life of people living with PD.
